# Inferring parturition and neonate survival from movement patterns of female ungulates: a case study using woodland caribou

**DOI:** 10.1002/ece3.785

**Published:** 2013-09-23

**Authors:** Craig A DeMars, Marie Auger-Méthé, Ulrike E Schlägel, Stan Boutin

**Affiliations:** 1 Department of Biological Sciences, University of AlbertaEdmonton, AB, T6G 2E9, Canada; 2 Department of Mathematical and Statistical Sciences, University of AlbertaEdmonton, AB, T6G 2G1, Canada

**Keywords:** Animal movement, GPS telemetry, fitness, calving, pregnancy, offspring survival, demographic rates, woodland caribou, *Rangifer*

## Abstract

Analyses of animal movement data have primarily focused on understanding patterns of space use and the behavioural processes driving them. Here, we analyzed animal movement data to infer components of individual fitness, specifically parturition and neonate survival. We predicted that parturition and neonate loss events could be identified by sudden and marked changes in female movement patterns. Using GPS radio-telemetry data from female woodland caribou (*Rangifer tarandus caribou*), we developed and tested two novel movement-based methods for inferring parturition and neonate survival. The first method estimated movement thresholds indicative of parturition and neonate loss from population-level data then applied these thresholds in a moving-window analysis on individual time-series data. The second method used an individual-based approach that discriminated among three *a priori* models representing the movement patterns of non-parturient females, females with surviving offspring, and females losing offspring. The models assumed that step lengths (the distance between successive GPS locations) were exponentially distributed and that abrupt changes in the scale parameter of the exponential distribution were indicative of parturition and offspring loss. Both methods predicted parturition with near certainty (>97% accuracy) and produced appropriate predictions of parturition dates. Prediction of neonate survival was affected by data quality for both methods; however, when using high quality data (i.e., with few missing GPS locations), the individual-based method performed better, predicting neonate survival status with an accuracy rate of 87%. Understanding ungulate population dynamics often requires estimates of parturition and neonate survival rates. With GPS radio-collars increasingly being used in research and management of ungulates, our movement-based methods represent a viable approach for estimating rates of both parameters.

## Introduction

In the last 20 years, the analysis of animal movement has been a fundamental component of wildlife research and management. Movement analyses have been used to infer a broad range of animal behaviour and to assess the spatial dynamics of individuals and populations. For example, movement models based on step lengths, turning angles (the relative directional change in movement trajectory) and autocorrelation (the tendency to move in a similar direction or pattern) have yielded insights as to how animals move in heterogeneous landscapes (Johnson et al. 2002; Morales and Ellner 2002; Forester et al. 2007) and establish home ranges (Moorcroft et al. 2006; Börger et al. 2008; Moorcroft 2012). Specific behaviours such as foraging can be inferred using models that classify segments of movement paths based on the expected differences in movement characteristics of distinct behavioural states (Frair et al. 2005; Gurarie et al. 2009; Breed et al. 2012). The increasingly finer spatial and temporal resolution of GPS radio-telemetry data has facilitated an expansion in the variety and complexity of movement models (Schick et al. 2008; Smouse et al. 2010). However, the primary objectives for most movement studies remain similar: relating animal movement to environmental variation, behavioural states, or predictions of space use.

**Figure 1:**
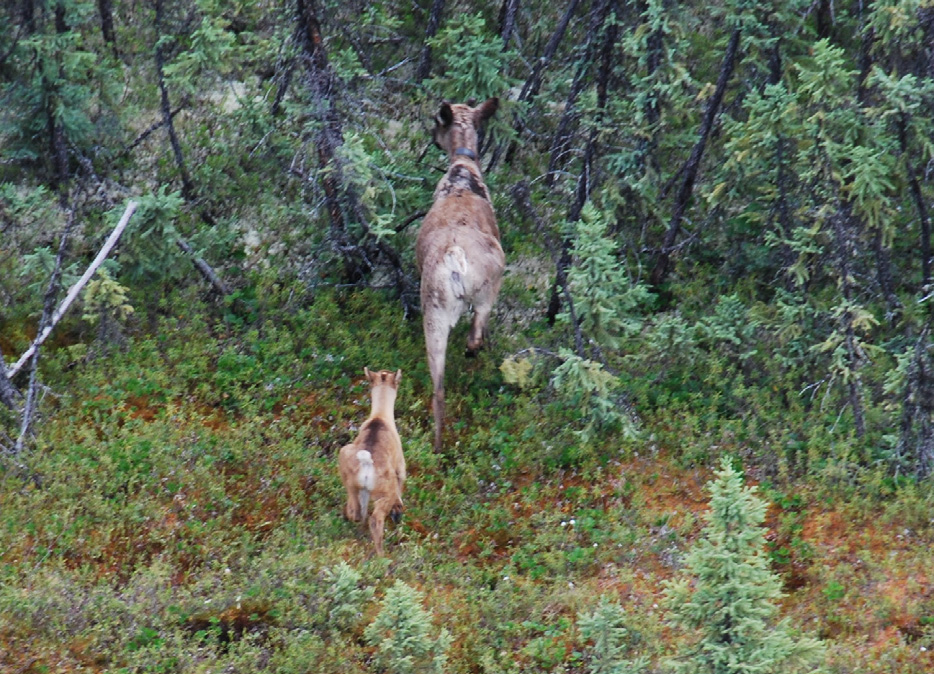
Female boreal caribou with a neonate (≤4 week old) calf in northeast British Columbia, Canada.

Here, we analyze animal movement data to infer components of individual fitness. Specifically, we develop and evaluate two methods for inferring parturition and survival of neonatal offspring (0–4 weeks of age) using movement data from female woodland caribou (*Rangifer tarandus caribou*; Fig. 1). For each parameter, we scale up individual predictions to estimate population rates, an important extension because parturition and neonate survival are components of calf recruitment, a key driver of ungulate population dynamics (Gaillard et al. 2000; Raithel et al. 2007; DeCesare et al. 2012).

Previous methods for assessing parturition in wild ungulates have included aerial surveys during the calving season (Whiting et al. 2012), serum progesterone tests on blood samples taken from captured animals (Wittmer et al. 2005), and vaginal implant transmitters (Powell and DelGiudice 2005; Barbknecht et al. 2011). Parturition has also been inferred by subjectively assessing for spatial clustering of GPS locations or changes in movement patterns (e.g., depressed daily movement rates for >3 days) but these visual approximation methods have not been rigorously validated (Bowyer et al. 1999; Ferguson and Elkie 2004; Long et al. 2009); but see Nagy 2011). Recently, Dzialak et al. (2011) used a general additive modelling approach to infer parturition from movement data of female elk (*Cervus canadensis*). Their correlative approach, however, resulted in a high rate of false-negatives. We expand on the idea of inferring parturition from female movement patterns by more explicitly modelling the movement process and objectively evaluating our methods across three data sets.

We further extend our analyses to develop a novel approach for estimating rates of neonate survival. Rates of neonate calf survival are usually determined by late spring aerial surveys or by radio-collaring newborn calves (Gustine et al. 2006; Barber-Meyer et al. 2008). By using only movement data of maternal females, we develop methods for estimating both parturition and survival that are potentially more cost-effective and less invasive to newborn offspring than traditional methods. Moreover, effective movement-based methods could be used to retroactively analyze historical radio-telemetry data sets to examine long-term trends in both rates.

We tested whether parturition status and neonate survival can be reliably inferred from female caribou movement patterns using population-based and individual-based methods. We predicted that calving events could be identified by a sudden and marked change – or break point – in normal female movement patterns, specifically a significant reduction in mean step length. For those females that calved, we predicted that movement rates would remain depressed as long as the calf was alive due to the relative immobility of the neonatal calf acting as a spatial “anchor”. Conversely, if the calf was lost during the neonatal period, we hypothesized that a second break point would be evident with female movement rates abruptly returning to pre-calving levels. Female movement patterns lacking a second break point would be indicative of calf survival through the neonate period. We limited our analysis to the neonate period because calf mobility after 4 weeks of age likely begins to approach adult movement rates – as evidenced by a sharp decline in bear predation of ungulate calves beyond this age (Zager and Beecham 2006; Barber-Meyer et al. 2008; Pinard et al. 2012) – thereby making breaks in movement patterns less discernible.

## Methods

### Caribou movement data

We developed and tested our two methods using GPS location data collected from reproductive-aged (≥3 years old) female caribou captured from four different caribou ranges near Fort Nelson, British Columbia, Canada. For method development, we used data from females captured in 2011 (*n* = 25) and 2012 (*n* = 2). These animals were fitted with Iridium GPS radio-collars (model G2110E, Advanced Telemetry Systems (ATS), Isanti, MN) programmed to acquire one GPS location (or fix) every two hours from April 15 – July 15 and once per day otherwise. For three-dimensional GPS locations (3D; see below), the mean horizontal measurement error of the collars was estimated to be 7.7 m (C. DeMars, unpublished data). Approximately half of the collars deployed in 2011 remained operational through two calving seasons. We partitioned the locations from these collars into two data sets, using 2011 data for method training (*n* = 24) and 2012 data for method testing (*n* = 15; 3 individuals unique from 2011 data). To further evaluate method performance, we used data from a study conducted in the same area during 2004 (*n* = 10). Individuals from this study were fitted with GPS radio-collars programmed to record locations at four-hour intervals continuously (models POSREC C600 or C900, Televilt/TVP Positioning AB, Lindesberg, Sweden; or model G2000, ATS, Isanti, MN). The mean horizontal measurement error associated with the 2004 collars was unknown.

All caribou were captured and handled in accordance with approved governmental and institutional animal care protocols (British Columbia Resource Inventory Committee 1998; University of Alberta animal use protocol # 748/02/12). Individual females in each data set were captured by aerial net-gunning from a helicopter during the mid- to late-winter months (January – March). Captured animals were physically restrained during collar attachment and were not anesthetized.

For the 2011 data, the pregnancy status of all females is known from blood serum progesterone tests performed on samples taken upon capture (pregnancy: ≥2.0 ng/mL; Prairie Diagnostic Services, Saskatoon, SK). We confirmed calving events by conducting weekly aerial surveys during the calving season. All collared females received at least two surveys and all individuals classified as pregnant on progesterone testing were observed with a calf. We estimated calving dates by visually estimating calf age based on a calf's size, relative mobility and pelage colour (Lent 1966). We corroborated this information by assessing for a significant drop in female movement rates (Nagy 2011). For a subset of 12 females that calved, we continued aerial surveys after calving to assess calf survival to 4 weeks of age. Females observed to have lost their calf on a survey were subsequently re-surveyed to confirm calf status. As none of these females were subsequently observed with a calf, we assumed that true calf survival status for this subset was known. For 2012 data, the pregnancy status of females is unknown. We therefore classified these females as either calf presence or absence by aerial survey with absence indicating either calf loss or non-pregnancy. For the 2004 data, pregnancy status is known from serum progesterone tests and calving events and calf survival to 4 weeks of age were confirmed by aerial survey.

To standardize data sets, we used a sampling rate of every four hours and limited the time-series to GPS locations taken from April 15 to June 30 inclusively – the calving season of caribou in our study area. We further rarefied the data by excluding locations with low accuracy [e.g. fixes <3D; <3% of all locations; (Frair et al. 2010)] and locations from 10:00 to 18:00 h on dates of aerial surveys to remove step lengths potentially influenced by helicopter-associated disturbance. After rarefaction, the mean per-collar fix rate (number of successful fixes/number of attempts; Frair et al. 2010) was 95% (range: 86–98%) in 2011, 96% (range: 92–99%) in 2012, and 77% (range: 49–90%) in 2004. To ensure a consistent time interval across the time-series of each individual, we assigned a missing value to locations removed by our rarefying procedures and those associated with occasional missed GPS fixes by the radio-collars. In all analyses, we used only step lengths calculated from successive GPS locations (i.e. steps initiated or ending at a missing value were excluded).

### Population-based method

**Figure 2:**
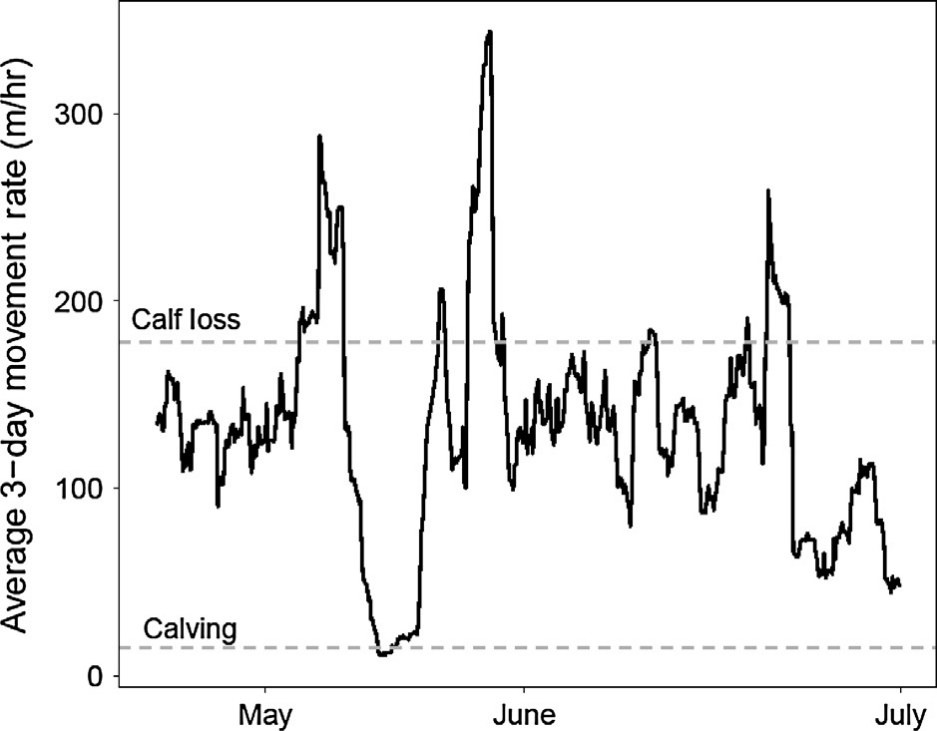
Analysis of movement patterns of female woodland caribou using the population-level method to infer parturition and offspring survival status. In this example, the female is predicted to have calved in the middle of May with the calf lost approximately 1 week later.

For the first method, hereafter referred to as the population-based method (PBM), we developed population-level thresholds of three-day average movement (TDAM) rates (m/h) to predict calving events and calf survival (Fig. 2; Appendix S1). To establish each threshold, we used empirical distributions of TDAM rates derived from step lengths contained within the 2011 data set. We used the calving and calf loss thresholds in a three-day moving window analysis applied to the time-series of movement data for individual caribou across all data sets. During preliminary analyses, we considered alternate time intervals (e.g. one- and two-day windows) but these did not improve method performance.

**Figure 3:**
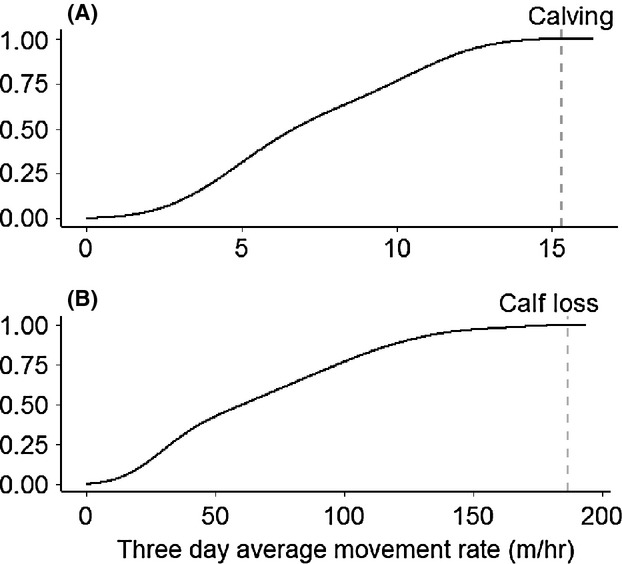
Cumulative distribution functions (CDFs) used to calculate the calving (A) and calf loss (B) thresholds for the population-based method (PBM). Grey dotted lines represent 3 day average movement rates at the 99.9% quantile of each CDF.

To estimate a calving threshold, we first created a distribution of TDAM rates using step lengths taken from the first 3 days post-calving of females known to have a calf surviving at least 1 week (*n* = 10). We converted this empirical distribution into a smooth kernel density estimate (KDE) using the ‘density’ function in R 2.15.0 (R Core Team, 2012). This KDE represents an estimate of the population-level distribution of possible TDAM rates of females with calves less than 3 days old. We then transformed the KDE into a cumulative distribution function (CDF), which represents the proportion of the population expected to move at or below a given rate (Fig. 3). We used the 99.9% quantile of the resultant CDFs as a calving threshold. In the subsequent moving window analysis performed on individual time-series data, females with TDAM rates dropping below this threshold would be indicative of calving.

We applied a similar approach to establish a calf loss threshold using step lengths from two to 4 weeks post-calving of females with calves surviving to 4 weeks (i.e. calving date and calf survival confirmed by aerial survey – see above; *n* = 6). Prior to calculating TDAM rates, however, we first rarefied the data to exclude the top 1% of step lengths. We considered these movements to be abnormal, perhaps related to instances of predator avoidance, and their removal ensured TDAM rates more accurately reflected average movement behaviour over the time interval (3 days), thereby increasing the method's sensitivity to correctly identify instances of true calf loss. As for previous rarefaction procedures, steps removed were assigned a missing value. After rarefying, we calculated a CDF of TDAM rates using the same procedures as per the calving threshold. We used the 99.9% quantile of this CDF as a calf loss threshold, which represents the maximum expected TDAM rate of females with calves up to 4 weeks old. In the subsequent moving window analysis, we applied the same rarefying procedure to post-calving steps of females known to have calved, then calculated TDAM rates and identified instances of calf loss when TDAM rates first exceeded the calf loss threshold.

As the threshold values are dependent on the individuals sampled, we evaluated the robustness of PBM predictions to variation in threshold specification. We calculated 95% bootstrap confidence intervals for both the calving and calf loss thresholds by iteratively resampling with replacement individual caribou used to estimate each threshold (*n* = 1000 iterations for each threshold). Using the upper and lower bounds of these confidence intervals, we repeated the moving window analysis on all data sets to determine the extent to which predictions changed depending on the threshold value used.

### Individual-based method

**Figure 4:**
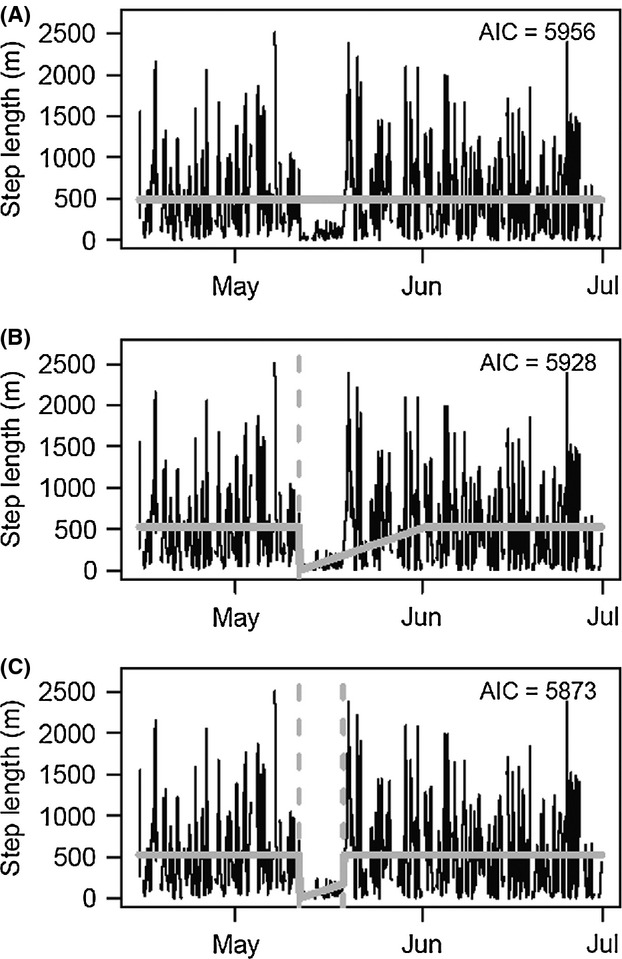
Movement models used in the individual-based method to infer parturition and offspring survival status in female woodland caribou. The black line represents the movement pattern of a female that gave birth ∼ May 11 then lost her calf ∼ May 19 [note: each graph represents the same movement data]. Solid grey lines represent the scale parameter of the exponential distribution, interpreted here as the mean step length. Vertical dashed lines represent estimated break points in the time series. A constant scale parameter with no break point indicates no calving (A), a single break point indicates a female with a calf that survived (B), while two break points indicates a female that calved then subsequently lost the calf (C). In this instance, the model with two break points (C) was the best fit to the data.

For the second method, referred hereafter as the individual-based method (IBM), we developed *a priori* movement models representing the three states of females during the calving season (i.e., did not calve, calved and calf survived to 4 weeks, and calved with subsequent calf loss; Appendices S2–S3). All models assume step lengths are exponentially distributed and differ only in their scale parameter – the only parameter of the exponential distribution – which in our analysis can be interpreted as mean step length (Fig. 4). For the model representing females that do not calve (*M*_*0*_), the scale parameter (*b*_*0*_) remains constant over time. For the model representing females with calves surviving to 4 weeks (*M*_*1*_), the scale parameter abruptly drops at calving from its pre-calving constant (*b*_*1*_*)*, creating a break point (*BP*_*1,c*_). The scale parameter then linearly increases with a slope of *b*_*1*_/*k*_*1*_, where *k*_*1*_ is the time, defined in number of steps, required for the calf to achieve adult movement rates. For the model representing females losing calves (*M*_*2*_), there is an abrupt change in the slope of the linear increase post-calving, creating a second break point (*BP*_*2,l*_) after which the scale parameter immediately recovers its pre-calving value (*b*_*2*_). The models therefore differ in their number of parameters to estimate: *M*_*0*_ has one – *b*_*0*_; *M*_*1*_ has three – *b*_*1*_, *k*_*1*_ and *BP*_*1,c*_; and *M*_*2*_ has four – *b*_*2*_, *k*_*2*_, *BP*_*2,c*_ and *BP*_*2,l*_.

We discriminated among models using Akaike's Information Criterion (AIC) with the best model being the one with the lowest AIC value. Prior to model fitting, we removed the top 1% of step lengths from the whole time-series, which contrasts with the PBM where the rarefaction is applied only to post-calving steps. This *a priori* rarefaction was necessary to ensure that the three IBM movement models were evaluated against the same data set. We estimated all model parameters using an approximation of maximum likelihood estimation (Appendix S2) and applied the following constraints to each parameter. First, the scale parameters of the exponential distribution (*b*_*0*_*, b*_*1*_*,* and *b*_*2*_) by definition should be greater than zero. Second, we constrained the values of *k* to fall between three and 6 weeks of age because, as noted earlier, within this interval calf movement rates likely approach adult rates. Third, we constrained break points to be a minimum of 24 step lengths away from either end of the time series and from one another. This constraint allows for a sufficient number of observations for each movement phase to accurately estimate parameters and subsequently discriminate among models.

### Evaluating method performance

To evaluate predictive performance for each method, we calculated measures of sensitivity (proportion of true positives correctly identified), specificity (proportion of true negatives correctly identified) and accuracy (overall rate of true predictions). As both methods yield predictions of calving and calf loss dates (see Appendix S1 and S3), we also evaluated whether predicted dates fell within the appropriate aerial survey interval (e.g., estimated calving date was after a female was seen without a calf and before the same female was seen with calf on a subsequent survey). Because management of ungulates often relies on population-level estimates of parturition and survival (Gordon et al. 2004), we summed individual predictions to estimate rates of parturition and neonate survival. We compared these predicted rates to the true sample rates, which in turn are estimates of population rates for parturition and neonate survival. This comparison explicitly evaluates each method for any directional bias (e.g. under- or over-estimating neonate survival; Altman and Royston 2000).

We also evaluated the effects of data quality on method performance by conducting post-hoc sensitivity analyses where we reduced fix rate and simulated data gaps (Appendix S4). Because parturition predictions for each method remained unchanged in all analyses, we focus primarily on neonate survival. For each analysis, we used all individuals from the 2011 and 2012 data sets whose parturition and neonate survival statuses were correctly predicted by both methods and had fix rates >90%. To assess the effect of fix rate, we randomly subsampled within each individual to simulate fix rates of 90% down to 60% in 5% increments. To evaluate the effect of data gaps, we randomly removed intervals of one day, two non-consecutive days and two consecutive days from the post-calving period within the original time-series of each individual female. We further assessed the interacting effects of fix rate and data gaps. For all analyses, we ran 30 simulations across the data set of 13 individuals and calculated the mean accuracy rate (the percentage of correct predictions) for each method.

All analyses were performed with R 2.15.0 (R Core Team, 2012) and detailed descriptions of our R code can be found in Appendices S1 and S3.

## Results

### Parturition status

Both methods predicted calving events with near certainty (Table 1). For the PBM, we calculated a calving threshold of 15.3 m/h (Fig. 3a; sample range of TDAM rates 3 days post-calving: 3.78, 11.35 m/h) which correctly discriminated among females that calved and non-pregnant females in 2011 and 2004 (*n* = 34). This threshold also correctly predicted the calving status of the six females visually confirmed to have calved in 2012 (pregnancy status is unknown for this data set). Further, all estimated calving dates fell within the appropriate aerial survey interval across data sets. The PBM was relatively robust to changes in the threshold value as no predictions changed when using the upper 95% CI value (18.8 m/h) and only one female was misclassified when using the lower 95% CI value (13.2 m/h).

**Table 1:** Parturition and calf survival status predicted by the population-based (PBM) and individual-based (IBM) methods

			Correct predictions		Correct interval	
				
Year	Status	Known Status: Number	PBM	IBM	PBM	IBM
2011	Calving	Pregnant, calved: 19	19	18	19	18
		Not pregnant: 5	5	5	–	–
	Calf survival	Survived: 8	6	6	–	–
		Lost^1^: PLM = 4; IBM = 3	3	3	3	3
2012^2^	Calf presence	Confirmed calved: 6	6	6	6	6
		No calf: 9	6	8	–	–
	Calf survival	Survived: 4	4	4	–	–
		Lost: 2	2	2	2	2
2004	Calving	Pregnant, calved: 9	9	9	9	9
		Not pregnant: 1	1	1	–	–
	Calf survival	Survived: 5	4	2	–	–
		Lost: 4	2	3	2	2

1 The total number of calves known to be lost is one less for IBM as we excluded the female misclassified as not calving.

2 For 2012, pregnancy status is unknown therefore a status of no calf could indicate either not pregnant or calved and subsequently lost before the calf was observed.

The IBM correctly predicted calving status for 33 of 34 females across the 2011 and 2004 data sets and all six females confirmed to have calved in 2012. Estimated calving dates all fell within the appropriate survey interval. One female in 2011 was misclassified as not calving when in fact this individual was pregnant and observed with a calf. This female had the movement path with the smallest sample of step lengths in the 2011 data set (*n* = 331; range: 331–438).

Assessing sample rates of parturition, the PBM correctly estimated a rate of 19/24 for 2011 while the IBM produced an estimate of 18/24. Both methods correctly estimated a rate of 9/10 for 2004 and produced identical estimates of 12/15 for 2012 where the true sample rate is unknown.

### Neonate survival status

The two methods differed more in the ability to predict calf survival to 4 weeks of age (Tables 1 and 2). For the PBM, we calculated a calf loss threshold of 186.5 m/h (Fig. 3b sample range of TDAM rates: 12.9, 178.6 m/h), which correctly predicted the survival status of nine of 12 calves in 2011, all six calves visually observed in 2012, and six of nine calves in 2004. For calves correctly predicted as lost, all estimated loss dates fell within the correct survey interval. Because both methods had high predictive power for calving, we further examined calf survival for all females predicted to have calved in 2012. Given this assumption, the PBM correctly predicted nine of 12 calves, misclassifying three lost calves. The PBM was less robust to changes in threshold specification for calf loss compared to calving (Table 2). While the lower 95% CI value (153.0 m/h) minimally changed values of sensitivity – the proportion of lost calves correctly identified – and specificity– the proportion of surviving calves correctly identified –, the upper 95% CI value (249.5 m/h) underestimated calf loss.

**Table 2:** Sensitivity (the proportion of lost calves correctly identified), specificity (the proportion of surviving calves correctly identified) and accuracy (the proportion of correct predictions) of the population-level (PBM) and individual-based (IBM) methods for predicting calf survival across all data sets. For the PBM, the estimated threshold value (186.5 m/h) and the bounding values of its 95% bootstrap confidence interval are shown. For 2012 data, we assumed the predicted calving status was true and therefore included all females predicted to have calved

Performance measure	Data set	PBM			IBM

		153.0 m/h	186.5 m/h	249.5 m/h	
Sensitivity	2011 (*n* = 4[3])^1^	1.0	0.75	0.25	1.0
	2012 (*n* = 8)	0.63	0.63	0.38	0.88
	2004 (*n* = 4)	0.75	0.50	0.25	0.75
Specificity	2011 (*n* = 8)	0.63	0.75	1.0	0.75
	2012 (*n* = 4)	1.0	1.0	1.0	1.0
	2004 (*n* = 5)	0.80	0.80	1.0	0.40
Accuracy	2011 (*n* = 12)	0.75	0.75	0.75	0.82
	2012 (*n* = 12)	0.75	0.75	0.58	0.92
	2004 (*n* = 9)	0.78	0.67	0.67	0.56

1 The total number of calves known to be lost in 2011 is three for IBM as we excluded the female misclassified as not calving.

The IBM correctly predicted the survival status of nine of 11 calves in 2011 – excluding the female misclassified as not calving –, all six calves visually observed in 2012, and five of nine calves in 2004. For all females predicted to have calved in 2012, the IBM correctly predicted the survival status of 11 of 12 calves, misclassifying one lost calf. For calves correctly predicted as lost, seven of eight estimated loss dates fell within the appropriate survey interval.

In general, the IBM had higher rates of sensitivity and specificity than the PBM (Table 2). As a result, the IBM had higher accuracy (78%) than the PBM (73%) across all data sets. Performance of both methods decreased in 2004. Notably, this data set had a higher number of missing observations resulting in a lower mean number of steps per caribou (*n* = 283, range: 119–374) compared to the 2011 (*n* = 412; range: 331–438) and 2012 (*n* = 423; range: 394–446) data sets. With the 2004 data excluded, accuracy was 87% for the IBM and 75% for the PBM.

For assessing sample rates of calf survival, we pooled data across years because of small per-year sample sizes. The IBM estimated 13 surviving calves (true survival = 17/33 calves) while the PBM estimated 20. Considering only 2011 and 2012 data (true survival = 12/24 calves), the IBM estimated 11 surviving calves while the PBM estimated 14.

### Effects of data quality

We used all correctly predicted individuals from 2011 (*n* = 7) and 2012 (*n* = 6) for assessing the sensitivity of neonate survival predictions to data quality. In general, the PBM was more robust to decreasing data quality (Appendix S4). With decreasing fix rate, PBM mean accuracy was >90% until fix rates were <80% while IBM mean accuracy was <90% with fix rates <90% (Appendix S4). Data gaps had less effect than fix rate. When one day, two non-consecutive days and two consecutive days were removed, PBM mean accuracy remained >98% while IBM mean accuracy was slightly lower (88% -94%). When fix rate was interacted with data gaps, method performance was primarily dictated by fix rate with data gaps only slightly decreasing mean accuracy compared to fix rate alone. In all analyses, the majority (>95%) of incorrect predictions resulted from surviving calves being misclassified (i.e. neonate survival was underestimated).

## Discussion

Extracting information from animal movement data has been an active area of research for the past two decades but insights gained have been primarily restricted to understanding animal space use and the underlying behavioural processes driving these patterns (McLoughlin et al. 2010; Smouse et al. 2010). We expanded the scope of information to be gained from animal movement patterns by developing two quantitative methods to infer rates of parturition and neonate offspring survival, both components of individual fitness and often key drivers of population dynamics in wild animals (Stirling et al. 1999; Mahoney and Schaefer 2002; Ogutu et al. 2010). Our results demonstrate that both methods can yield highly accurate estimates of parturition rates and, when applied to high quality data obtained by modern GPS radio-collars, good estimates of neonate survival using the IBM.

### Parturition status

As predicted, a sudden and sustained drop in movement rate was indicative of parturition. While other studies have similarly suggested that parturition is correlated with depressed movement rates in ungulates (Poole et al. 2007; Long et al. 2009; Brook 2010), we validated two objective and quantitative methods for determining parturiency from time-series movement data. Both methods were highly accurate (>97%) in predicting parturition and provided appropriate predictions of parturition dates. Moreover, both methods had extremely low false-negative rates (PBM = 0%; IBM < 3%), which contrasts with the high false-negative rate (46%) that confounded the modelling approach of Dzialak et al. (2011; see Table 3 therein) for inferring parturition in elk.

The PBM performed slightly better for predicting parturition, likely due to its increased sensitivity to short duration changes in movement rates. By only considering movements within a three-day moving window, the PBM may detect parturition even in instances where offspring are lost shortly after birth. In contrast, the IBM considers the entire time-series to calculate likelihoods and discriminate among models. For this reason, it is critical that the time-series be restricted to the expected parturition period of the species because detecting differences in movement rates will become increasingly difficult as the volume of movement data outside of the parturition period increases. Nevertheless, even with this requirement, if parturition is close in time to offspring loss, only a small number of data points will be available to create significant changes in the likelihoods and differences among models. In such instances, the non-parturient model will be the most parsimonious fit to the data because movement rates will have changed relatively little when the entire time-series is considered. We attempted to partially overcome this limitation by constraining parturition and offspring loss break points to be at least 24 time steps apart. This constraint explicitly inhibits the ability of the IBM to detect parturition events where offspring loss is within 4 days of birth, or slightly longer if the data have significant gaps. However, given that the IBM still correctly predicted the parturition status of 33 out of 34 females, offspring losses occurring shortly after birth seem to constitute a small proportion of overall offspring losses, at least for the woodland caribou we monitored.

Both methods likely provide more accurate and cost-effective estimates of true parturition rates than traditional methods such as aerial surveys using radio-telemetry. When considering our 2012 data, only six of 15 females were confirmed to have calved by aerial survey whereas both movement-based methods estimated 12 females to have calved. Nagy (2011) reported a similar finding, suggesting that aerial surveys of woodland caribou underestimated parturition rates by 12–19%. The high correlation of our methods with serum progesterone tests for pregnancy further validates the use of movement-based approaches for estimating population-level parturition rates.

### Neonate survival status

For predicting neonate survival, the IBM generally performed better, producing reasonably accurate estimates particularly when using the higher quality data of 2011 and 2012 (87% accuracy across both data sets). We attempted to improve the accuracy of both methods by incorporating information other than step length. We considered changes in turning angles and autocorrelation (Gurarie et al. 2009) as well as spatial information (e.g. net-squared displacement from calving site; Fryxell et al. 2008) but found these additional parameters to be uninformative for predicting neonate survival from caribou movement patterns.

At the sample level, predicted rates of neonate survival were close to true sample rates, particularly for the IBM. It is important to note, however, that predicted survival rates incorporate both correct and false predictions of individual survival. This method of comparing grouped predicted outcomes to grouped known outcomes – known as calibration (Altman and Royston 2000) – is necessary because of the difficulty in translating evaluative measures of individual-based binary classification tests (e.g. accuracy) into estimates of uncertainty for population rates. Calibration yields more explicit insight into potential directional biases associated with each method. Our comparison of predicted to known sample rates of survival suggests that neonate survival is overestimated by the PBM and slightly underestimated by the IBM though a more rigorous evaluation of potential bias would require additional years of data.

The better performance of the IBM was likely due to its predictive ability being less influenced by inter-individual variability. By assigning to each individual a distinct baseline for movement rates, prediction by the IBM is confined to evaluating information solely within each individual. In contrast, the PBM compares individual movement rates to population-level thresholds and is thus contingent on variation within the population. This difference becomes increasingly important as the magnitude of movement variability among individuals increases. Variability in movement rates among females likely increases with offspring age such that the variability among females with 4 week old offspring is much greater than the variability among females in the first few days post-partum (Testa et al. 2000; Odonkhuu et al. 2009). With increasing variability, selecting a threshold that is adequate for all individuals becomes increasingly difficult, particularly in populations where individual personalities may vary widely from “slow” to “active” (Sih et al. 2004; Boon et al. 2008). If a threshold indicative of calf loss is too low, “active” mothers and offspring may exceed the threshold, resulting in an overestimation of offspring loss. Conversely, if a threshold is too high, females with normally “slow” movement rates may not exceed the threshold after offspring loss, resulting in an overestimation of offspring survival. This latter scenario was illustrated by the low sensitivity values recorded when we used the upper bound of our threshold's 95% confidence interval for prediction. Because high inter-individual variation in movement rates is prevalent in many animal populations (Odonkhuu et al. 2009; Olson et al. 2010; Mueller et al. 2011), individual-based approaches such as the IBM will be preferable for predicting neonate survival in most instances.

Misclassification of calf status may also be influenced by the agent of mortality. In most ungulate populations, predation is the primary cause of mortality in neonatal offspring with a relatively small proportion lost to disease and other natural causes (Gustine et al. 2006; Barber-Meyer et al. 2008; Carstensen et al. 2009). In predation events where the predator represents a threat to the female as well as her offspring (e.g. wolves [*Canis lupus*] for ungulates), the change in maternal movement should be sudden and sustained, creating a break point that is clearly evident. Conversely, if offspring are lost to natural causes or to a predator that does not directly threaten the female, then a break point may be less discernible, particularly if the female remains in the area for a few days before slowly moving away. This latter scenario could result in a false prediction of offspring survival. In our study, we did not have information regarding the true causes of calf mortality to directly assess this potential effect; however, determining whether female movement patterns contain information regarding the possible cause of offspring mortality represents an area for future research.

Due to the analytical framework of both methods, false predictions of offspring survival can result when offspring mortalities occur near the end of the time-series data. For the IBM, this situation results in a small number of post-loss steps being available to differentiate between the models of offspring survival and offspring loss. Moreover, pre-loss and post-loss movement rates will be similar because offspring movement rates are approaching adult rates at this time. Because the IBM uses the entire time-series to compare models, parsimony may favour the model of offspring survival. With the PBM, if offspring loss occurs on the last day of the time-series (i.e. when the calf is 3 weeks and 6 days old), the number of data points post-loss may be insufficient to raise the average of the three-day moving window above the threshold value indicative of calf loss. Overall, when estimating survival rates at the population level, any potential bias resulting from the timing of offspring loss should be small unless true offspring losses are skewed toward the end of the neonatal period (e.g., near 4 weeks of age for caribou).

Data quality was the most significant factor influencing the ability to predict offspring survival. Increasing gappiness in the time-series data negatively affected predictive performance of both methods. On average, the 2004 data set had a 30% reduction in the number of steps available for estimation compared to the other data sets, with some individuals missing complete days of data. Data quality affected each method differently. For the IBM, specificity was reduced because consecutive missed steps could affect the assumption that offspring movement rates increase linearly post-partum. If movement rates after the data gap significantly differed from movement rates before the gap, then a second break point in the time-series could be created regardless of true offspring survival status, resulting in overestimation of offspring loss (see also Appendix S3). For ungulates in particular, data gaps would be especially problematic in the 2–4 week interval post-partum when offspring movement rates can change substantially over a short interval (Testa et al. 2000; Odonkhuu et al. 2009). Alternatively, if the PBM is used, data gaps could result in a small number of data points within the three-day moving window, increasing the probability of sampling error. As a result, estimated movement rates within these data-deficient windows may differ significantly from true values. Because of these issues, data quality needs to be assessed prior to the application of movement-based methods for estimating offspring survival rates. For data sets with fix rates of <90%, estimates of offspring survival may be unreliable, particularly when gaps spanning more than a day are present. However, with fix rates and location accuracy continuing to improve in modern GPS radio-collars (Frair et al. 2010), issues related to data quality should be less problematic with more recent data sets.

The better performance of both methods with the 2011 and 2012 data sets may have been influenced by the carryover of some individuals from 2011 into 2012, creating an issue of non-independence. We note, however, that for the PBM only three of the individuals used to establish the calf loss threshold were included in the 2012 data set and all three had different known statuses in 2012 (e.g. calf survived in 2011, calf lost in 2012). Further, of the 12 females predicted to have calved in 2012 and therefore considered for further analysis of calf survival, only five were carried over from 2011 and of these five, four had different known statuses between years. Based on this information, any non-independence issues likely had minimal impact on our overall results and inferences.

## Conclusion

With GPS radio-collars increasingly being used in the study and management of animal populations (Hebblewhite and Haydon 2010), movement-based methods are a viable approach for estimating rates of parturition and offspring survival in ungulates. The methods described here could easily be adapted to other species though the reliance on movement data of independent individuals precludes species that aggregate during the parturition season and therefore are influenced by group movement dynamics [e.g. barren-ground caribou (Lent 1966)]. We further caution that non-independence of movement behaviour may result from abnormal weather events (e.g. deep snow); thus, we recommend inspecting the raw movement data prior to method application to assess for correlations between environmental conditions and sample-wide aberrations in movement patterns. Of the two methods tested, the IBM is more directly applicable as it does not require training data to establish *a priori* species-specific thresholds. For caribou, we found step length to be the most informative variable for determining parturition and offspring survival rates. For other species, incorporating additional movement information such as turning angle and autocorrelation into movement models may prove useful for identifying break points indicative of parturition and offspring loss.

## Supporting Information

**Appendix S1.** R Code for the Population-based Method.

**Appendix S2.** Likelihood Functions Used in the Individual-based Method.

**Appendix S3.** R Code for the Individual-based Method.

**Appendix S4.** Post-hoc Analyses of the Effects of Data Quality on Method Performance.
