# Supplementary material for: Inferring parturition and neonate survival from movement patterns of female ungulates: a case study using woodland caribou
Source: Ecol Evol. 2013 Sep 23;3(12):4149–60. doi: 10.1002/ece3.785 (PMC3853560; doi:10.1002/ece3.785)
Supplement: Supplementary file 1 [file ece30003-4149-SD1.docx]

**Appendix 1**: R Code for the Population-based Method

Here we provide and explain the two central R (R Development Core Team, 2012) functions that we developed for the population-based method (PBM). As described in the main paper, the analysis is performed on three-day average movement rates. For the remainder of this appendix, we will call these three-day average movement rates simply “TDAM rates”.

*Threshold Establishment*

To detect calving events and, if applicable, calf loss events, we tested whether TDAM rates fall below or exceed, respectively, specific threshold values. We determined these threshold values using a training data set. The makeThresh() function we provide here can be used to establish both the calving and the calf loss threshold by applying it to the corresponding subset of the data. For the calving threshold, we used data from the first three days post-calving of females known to have a calf surviving at least one week. For the calf loss threshold, we used data from two to four weeks post-calving of females with calves known to have survived to four weeks of age.

The data needed as input to the makeThresh() function is a matrix of regular movement rates, where columns correspond to individual females. To calculate such movement rates, we first generated a time-series of step lengths, defined as the distance between consecutive GPS locations. This time-series has to be regular, meaning all calculated step lengths must span the same time interval (in our case, every four hours). Because GPS locations are often not recorded at exact times, we allowed a deviation up to a tolerance of one tenth of the time interval (i.e. 24 minutes). For missing GPS locations (e.g., missed fixes by the radio-collars), a value of “NA” is assigned thereby ensuring there is a record (i.e. row) for every fourth hour in the time-series. Note that every missed GPS location will result in at least two missed step lengths. After generating the time-series of step lengths, we divided each step length by the length of the time interval to obtain movement rates. We transformed these movement rates into a matrix where each column contains the movement rates of an individual female.

The makeThresh() function contains the option to rarefy the data prior to calculating a specific movement threshold. The default of this option is no rarefaction. As noted in the main text, we rarefied the two-to-four week post-calving data used to calculate the calf loss threshold by removing the top 1% of step lengths, movements we considered abnormal and possibly associated with instances of predator avoidance. Removal of these outliers increases the sensitivity of the PBM to correctly identify instances of true calf loss. Steps removed by rarefaction are assigned a value of “NA”.

After inputting the movement rate matrix and specifying whether to rarefy the data, the makeThresh() function generates TDAM rates for each time-series by calculating a moving average with window size of three days. To calculate TDAM rates, we use the rollapply() function from the R package ‘zoo’ (Zeileis & Grothendieck 2005). Because missing data points are included as “NA” in the time-series, the moving average is calculated from the available data points within the three-day window (as opposed to calculating a moving average for a fixed number of available data points). Note that for the calving threshold data, this analysis results in exactly one TDAM rate per female. For the calf loss threshold, multiple TDAM rates are generated per female because the data considered spans 14 days (i.e. from two to four weeks post-calving).

Calculated TDAM rates are then pooled together into one vector to generate an empirical distribution of possible TDAM rates within the considered time frame. From this vector, the makeThresh() function estimates a probability density function for TDAM rates using the density()function in R. To calculate the corresponding cumulative distribution function (CDF), which requires integrating the density, the resulting output from density()is converted into a function via the approxfun() function in R. After calculating the CDF, we used the 99.9% quantile of the CDF as the threshold value. We chose this particular quantile because we wanted to obtain the largest TDAM rate possible under the estimated probability distribution. Many analytical probability distributions have a CDF that asymptotes at 1.0; hence, we chose a quantile very close to 1.0. However, practically, our distribution of TDAM rates has a finite domain so it may also be possible to use the 100% quantile. The obtained quantile is set as the threshold and returned as the value of the function.

The makeThresh() function provides the option to plot the histogram of TDAM rates, together with the estimated probability density function. A vertical bar indicates the threshold value.

makeThresh <- function(moveRates, timeInt, rare=F, draw=F){

if (rare==T){

rarIndex <- apply(moveRates, 2, function(x) quantile(x, probs=0.99, na.rm=T))

for (i in 1:ncol(moveRates)) moveRates[moveRates[,i] > rarIndex[i],i] <- NA

}

rollAverage <- rollapply(moveRates, 3*24/timeInt, mean, na.rm=T, by.column=T)

rollPool <- as.vector(rollAverage)

rollDensity <- density(rollPool)

densityFun <- approxfun(rollDensity$x, rollDensity$y, yleft=0, yright=0)

y <- seq(1, max(rollPool)+20, 0.1)

rollCumu <- rep(NA, length(y))

for (i in 1:length(y)) rollCumu[i] <- integrate(densityFun, -Inf, y[i], stop.on.error=F)$value

quant <- 0.999

threshold <- y[which(rollCumu >= quant)[1]]

if (draw==T){

hist(rollPool, 50, freq=F, xlim=c(0,threshold+10), xlab="TDAM mean movement rates", main="Histogram, Density and Threshold")

lines(rollDensity, col='red', lwd=2)

abline(v=threshold, lwd=2, col='blue')

}

return(threshold)

}

*Data Analysis*

To perform the PBM using the thresholds established above, we developed the function getStatus(). This function analyzes data for one individual cow, and therefore has to be applied to each individual separately (e.g., within some wrapper).

The data input for this function is a list, which contains at least three elements. An element named “MR” is a complete time-series of regular movement rates. This time-series should span the parturition and neonate season of the species under consideration. An element named “tp” is a vector of dates and times – in the R class POSIXct format – at which the movement rates were measured. Because of the format of MR, the vector tp should contain regular times, up to a certain tolerance (as explained in the previous section). An element named “interval” is a numeric value indicating the time interval spanning one step (here, 4). In addition to the data list, the calving threshold and the calf loss threshold need to be passed to the function. It is important that the unit for the movement rates is the same for threshold generation and data analysis (e.g., meters/hour).

After inputting the data list and threshold values, the getStatus() function first converts the time-series of movement rates into a series of TDAM rates, analogous to the previous section. Next, the calving status is determined by querying the series of TDAM rates for values that fall below the calving threshold. For the first TDAM rate falling below the calving threshold, we set the calving date as the date and time of the last step within the corresponding three-day window. If a calving event is detected, the data is further analyzed to determine calf survival to four weeks of age. Starting from the previously estimated calving date, a four week subset of MR is isolated and then rarefied to remove outliers, defined here as the upper 1% of movement rates (see main paper for rationale). After rarefaction, TDAM rates are again calculated and searched for values that exceed the calf loss threshold. For the first TDAM rate exceeding the calf loss threshold, the estimated date of calf loss is the date and time of the last step in the corresponding three-day window. If no TDAM rate exceeds the threshold, the calf is predicted to have survived to four weeks of age.

The getStatus() function returns a data frame that contains the following information. “Calved” is logical and indicates whether a female calved. “Calving Date” returns the estimated date of calving and “NA” if the female was predicted to not have calved. “Lost” is logical and indicates whether a calf was lost. If there was no calf, “Lost” is set to “NA”. “LostDate” returns either the estimated date of calf loss or “NA” if the calf survived.

getStatus <- function(movF, threshCalf, threshLoss){

meanMR <- rollapply(movF$MR, 3*24/movF$interval, mean, na.rm=T)

calved <- any(meanMR < threshCalf)

calfIndex <- which(meanMR < threshCalf)[1] + 17

calfDate <- movF$tp[calfIndex]

if (!is.na(calfIndex)){

wk4 <- calfIndex + 28*24/movF$interval

postCalfMR <- movF$MR[calfIndex:wk4]

postCalfDate <- movF$tp[calfIndex:wk4]

rarIndex <- quantile(postCalfMR, probs=0.99, na.rm=T)

postCalfMR[postCalfMR > rarIndex] <- NA

meanPcMR <- rollapply(postCalfMR, 3*24/movF$interval, mean, na.rm=T)

calfstatus <- any(meanPcMR > threshLoss)

lossIndex <- which(meanPcMR > threshLoss)[1] + 17

lossDate <- postCalfDate[lossIndex]

}else{

calfstatus <- NA

lossDate <- NA

}

results <- data.frame(Calved = calved, CalvingDate = calfDate, Lost = calfstatus, LossDate = as.character(lossDate))

return(results)

}

An R script containing the two functions can be found on our website https://sites.google.com/site/babybou2013.

**References:**

Zeileis, A. and G. Grothendieck (2005). zoo: S3 Infrastructure for

Regular and Irregular Time Series. *Journal of Statistical Software*, 14(6),

1-27. URL <http://www.jstatsoft.org/v14/i06/>

R Core Team (2012). R: A language and environment for statistical computing. R Foundation for Statistical Computing, Vienna, Austria. ISBN 3-900051-07-0, URL <http://www.R-> project.org/.

**Appendix 2**: Likelihood Functions Used in the Individual-based Method

In this appendix we describe the likelihood functions for each of the three models of the Individual-based Method (IBM). These likelihood functions are for time-series of observed step lengths,$\vec{x}=(x_{t_{1}},x_{t_{2}},\ldots,x_{N})$, where$S=\left(t_{1},t_{2},...,t_{N}\right)\subseteq(1,2,...,T)$, and T is the full span of the time-series. A step length is defined as the distance between locations taken at regular time intervals. We handle missing locations by excluding the associated step lengths from the time-series and by keeping the associated true time indices. For example, if the step lengths at time t=2 and t=3 are missing from the time–series we have, $\vec{x}=(x_{1},x_{4},x_{5},\ldots,x_{T})$, $S=\left(1,4,5,\ldots,T\right)$, and thus$N<T$. The likelihood functions are appropriate for time-series with missing steps because we assume that the step lengths are independently distributed. See Appendix 3 for more details on the construction of step length time-series.

The three models assume that step lengths are exponentially distributed. This distribution is characterized by a scale parameter, *bt*, which varies in time according to the specified model. For the model of a female that does not calve, M0,the scale parameter remains constant through time. Thus *bt* = *b0* for all *t*. The likelihood function for M0 is:

$L_{0}(b_{0})=\prod_{t\in S} \frac{1}{b_{0}}\exp\left(-\frac{x_{t}}{b_{0}}\right)$. (1)

For the model of a female with a calf that survives, M1, the scale parameter, *bt*, varies as a function of three parameters: the pre-calving scale parameter, *b1*, the calving break point, *BP1,c*, and the number of steps required by the calf to attain an adult movement rate, *k1*. Specifically, we define *bt* for M1 by:

$b_{t}=\left\{\begin{matrix} b_{1} & fort\leq BP_{1,c} \\ \frac{(t-BP_{1,c})b_{1}}{k_{1}} & forBP_{1,c}<t\leq BP_{1,c}+k_{1} \\ b_{1} & fort>BP_{1,c}+k_{1} \end{matrix}\right.$ (2)

Thus, the likelihood function for M1 is:

$L_{1}(b_{1},k_{1},BP_{1,c})=\prod_{\begin{matrix} \left\{t\in S,t\leq BP_{1,c}\right\} \\ \left\{t\in S,t>BP_{1,c}+k_{1}\right\} \end{matrix}} \frac{1}{b_{1}} \exp\left(-\frac{x_{t}}{b_{1}}\right)\times\prod_{\left\{t\in S,BP_{1,c}<t\leq BP_{1,c}+k_{1}\right\}} \frac{k_{1}}{(t-BP_{1,c})b_{1} } \exp\left(-\frac{x_{t}k_{1}}{(t-BP_{1,c}) b_{1} }\right)$. (3)

For the model of a female with a lost calf, M2, the scale parameter, *bt*, is affected by parameters equivalent to those of M1 as well as the calf loss break point, *BP2,l*. Specifically, we define *bt* for M2 by:

$b_{t}=\left\{\begin{matrix} b_{2} & fort\leq BP_{2,c} \\ \frac{(t-BP_{2,c})b_{2}}{k_{2}} & forBP_{2,c}<t\leq BP_{2,l} \\ b_{2} & fort>BP_{2,l} \end{matrix}\right.$, (4)

where *BP2,l*≤ *BP2,c*+*k2*. Thus the likelihood for M2 is:

$L_{2}(b_{2},k_{2},BP_{2,c},BP_{2,l})=\prod_{\begin{matrix} \left\{t\in S,t\leq BP_{2,c}\right\} \\ \left\{t\in S,t>BP_{2,l}\right\} \end{matrix}} \frac{1}{b_{2}} \exp\left(-\frac{x_{t}}{b_{2}}\right)\times\prod_{\left\{t\in S,BP_{2,c}<t\leq BP_{2,l}\right\}} \frac{k_{2}}{(t-BP_{2,c})b_{2} } \exp\left(-\frac{x_{t}k_{2}}{(t-BP_{2,c}) b_{2} } \right)$, (5)

where *BP2,l*≤ *BP2,c*+*k2*.

To be able to compare the models using Akaike’s Information Criterion (AIC), we need maximum likelihood estimates (MLE) for the parameters of each model. This is easily achieved for M0 which has only one parameter to estimate. The analytical solution for the MLE of *b0* is:

$\hat{b}_{0}=\frac{1}{N}\sum_{t\in S} x_{t}$, (6)

where N is the number of step lengths included in the time-series $\vec{x}$. In contrast, models M1 and M2 have no known analytical solution for the MLE of its parameters. Thus, these models require simultaneous numerical maximization of the likelihood with respect to all parameters: $MLE=\mathop{\mathrm{argmax}}\limits_{b,k,BP} L(b,k,BP)$, where *BP*, *b* and *k* stand for their corresponding versions in models M1 and M2. Such maximization can be computationally intense. This is particularly true for our likelihood functions because the values of two important parameters, the calving and calf loss break points, are natural numbers and many of the fast optimization methods are inappropriate for discrete parameters. A precise numerical maximization of a likelihood with such discrete-value parameters can be achieved by profiling the likelihood. To do so, we first maximize the likelihood at all possible *BP* values and then chose the BP value that maximize the overall likelihood, $MLE=\mathop{\mathrm{argmax}}\limits_{BP} (\mathop{\mathrm{argmax}}\limits_{b,k} L(b,k,BP))$. To reduce part of the computational complexity, we alter the likelihood functions. For M1 the likelihood function becomes:

, (7)

where $\hat{b}_{1}=\frac{1}{N_{BP_{1,c}}}\sum_{t\in S} x_{t}$ and $N_{BP_{1,c}}$is the number of step lengths in the time-series $\vec{x}$ with $t\leq BP_{1,c}$. Thus, the approximation of the MLE uses the mean observed step length prior to the calving break point as our estimate of *b1* and maximizes eqn. 7 with respect to only the parameters BP1,c and *k1*: . A similar procedure is performed for M2. The altered likelihood function is:

, (8)

where *BP2,l*≤ *BP2,c*+*k2,* $\hat{b}_{2}=\frac{1}{N_{BP_{2,c}}}\sum_{t\in S} x{}_{t}$ and $N_{BP_{2,c}}$is the number of step lengths in the time-series $\vec{x}$ with $t\leq BP_{2,c}$. The approximation of the MLE in this case is found using: . For more details on how the MLE was approximated please see Appendix 3 for the R code and its explanation.

Similar to others (e.g., Gurarie *et al.* 2009), we mainly used an approximation of the MLE to reduce computational time and complexity. This approach is an important consideration for our method given that tens of thousands of break point combinations are evaluated for each individual. In addition to its computational efficiency, this approximation is also biologically relevant. We are interested in identifying the moment in the time-series when the female recovers her normal, pre-calving movement. As such, using only mean pre-calving movement to estimate *b1* and *b2* focuses the movement model on changes from the pre-calving movement rather than on averaging the overall movement. We have also performed the analysis using the true (full) likelihood functions (eqns. 3 and 5). This analysis produced similar results and the R code for the full likelihood is available on our website: https://sites.google.com/site/babybou2013.

**References:**

Gurarie, E., Andrews, R.D. & Laidre, K.L. (2009). A novel method for identifying behavioural changes in animal movement data. *Ecology Letters*, **12**, 395–408.

**Appendix 3**: R Code for the Individual-based Method

This appendix includes the four R (R Development Core Team, 2012) functions necessary to apply the individual-based method (IBM) to an individual movement trajectory and their explanation. The R scripts and other R related information can be found on our website: <https://sites.google.com/site/babybou2013>. As mentioned in the main text, the IBM has three models: M0 for a female that does not calve, M1 for a female with a calf surviving to four weeks, and M2 for a female losing a calf prior to four weeks of age. When taken together, the first three R functions calculate the negative log-likelihood (nll) of M1 and M2 and are used to numerically estimate the minimum negative log-likelihood (mnll) and the maximum likelihood estimate (MLE) of the parameters of these two models. The fourth function calculates the mnll, MLE, and Akaike’s Information Criterion (AIC) of the three models and does so by calling the previous functions and, in the case of the M0, by directly calculating the analytical MLE and mnll.

The four functions rely on similar data inputs. SL is a numeric vector containing the step lengths measured at a regular time interval. ti is an integer vector identifying the time, in number of steps, of the step lengths present in SL. SL and ti should be of the same length and cannot contain NAs. tp is a POSIXct (an R class for dates and time) vector that identifies the real date and time of the SL. If there are missing steps, these should be represented with NAs in tp; thus, tp will be longer than SL and ti. These three vectors together represent the movement path of one individual. For example, for the (x,y) locations {(0,0), (0,1), (0,3), (1,3), (7,3)} at time {(2012/01/01 00:00), (2012/01/01 04:00), (2012/01/01 08:00), (2012/01/01 16:00), (2012/01/01 20:00)} from a radio-collar programmed to record a GPS location every 4 hours, SL would be {1, 2, 6}, ti would be {1, 2, 5}, and tp would be{(2012/01/01 00:00), (2012/01/01 04:00), NA, NA, (2012/01/01 16:00), NA}. We recommend that the time-series only include the parturition and neonate season of the species under consideration. In our case using woodland caribou, we only included movement from April 15th to June 30th. Using the R function quantile(), we also assign as missing steps all step lengths greater than the 99% quantile of SL.

As explained in the main text and in Appendix 2, all models assume step lengths are exponentially distributed and differ only in their scale parameters, *b*. Using the R function dexp(), we calculate the log-likelihood of all models by summing the log of the exponential probability density function of step lengths given a scale parameter. Note that dexp() requires the input of the rate rather than the scale parameter. The rate parameter of the exponential distribution is simply the inverse of the scale parameter (Forbes *et al.* 2011).

For models M1 and M2, the time-series is divided into pre-calving and post-calving sections. M2 further divides the post-calving section into pre-calf loss and post-calf loss sections. Thus, for functions nll1() and nll2(), which calculate nll of M1 and M2 respectively, SL and ti are divided into subsections defined by the calving and calf loss break points. *Section a* represents the time-series up to and including the calving break point. *Section b* represents the time-series after the calving break point, where the female is with her calf. For M1, *section b* includes the rest of the time-series. However, for M2, *section b* only includes the time-series up to and including the calf loss break point. After this second break point, the rest of the time-series is in *section c*.

The first function, nllk(), calculates the nll of *section b*, the section of the time-series where the female is with her calf. The scale parameter during this section increases linearly with a slope of *b1*/*k1* or *b2*/*k2*, represented in the code by ba/k, where ba is the pre-calving scale parameter and k is the time, in number of steps, it takes a female with a calf that survives to recover pre-calving movement. After *k* steps, the scale parameter returns to its pre-calving value, ba, and remains at this value for the rest of the time-series. nllk() is used in both functions nll1() and nll2() to numerically estimate the mnll of *section b* of the time series and the MLEs of *k1* and *k2*.

nllk <- function(k, SLb, tib, ba, tiBP1){

bb <- (tib - tiBP1) * ba / k

bb[ bb > ba ] <- ba

nllb <- -sum(dexp(SLb, 1/bb, log=TRUE))

return(nllb)

}

The second function, nll1(), calculates the nll of M1. This function first divides the time-series in two sections using the BP1,c value contained in the object BP. For *section a*, the pre-calving section, the scale parameter, ba, is estimated by the mean of the observed step lengths for this section. The mnll of *section a* is calculated directly using ba. For *section b*, the post-calving section, the mnll is numerically estimated using nllk() and the R function optimize(). The nll of *section a* and *b* are added together to get the overall nll of M1.

nll1 <- function(BP, SL, ti, kc){
 SLa <- SL[1:BP]
 n <- length(SL)
 SLb <- SL[ (BP + 1):n]
 tib <- ti[ (BP + 1):n ]
 ba <- mean(SLa)
 mnlla <- -sum(dexp(SLa, 1/ba, log=TRUE))
 mnllb <- optimize(nllk, kc, SLb=SLb, tib=tib, ba=ba,

tiBP1=ti[BP])$objective
nll <- mnlla + mnllb
return(nll)

}

Using methods similar to nll1(), the third function, nll2(), calculates the nll of M2. The function first divides the time-series into sections using the BP2,c and BP2,l values contained in object BP. For *section a*, the pre-calving section, and *section c*, the post-calf loss section, the MLE of the scale parameter, object ba, is the mean of the observed step lengths of *section a*. The mnll of *sections a* and *c* are calculated directly using ba. As for nll1(), function nllk() is used to calculate the mnll of *section b*. The nll of all sections are summed to calculate the overall nll.

nll2 <- function(BP, SL, ti, kc){
 SLa <- SL[1:BP[1]]
 SLb <- SL[(BP[1]+1):BP[2]]
 tib <- ti[(BP[1]+1):BP[2]]
 SLc <- SL[(BP[2]+1): length(SL)]
 ba <- mean(SLa)
 mnlla <- -sum(dexp(SLa, 1/ba, log=TRUE))
 mnllc <- -sum(dexp(SLc, 1/ba, log=TRUE))
 mnllb <- optimize(nllk, kc, SLb=SLb, tib=tib, ba=ba,

tiBP1=ti[BP[1]])$objective
nll <- sum(mnlla, mnllc, mnllb)

return(nll)
}

The fourth function, mnll3M(), estimates the mnll and AIC of all three models and the MLEs of all parameters. In addition to SL, ti, and tp, mnll3M() requires the input of two other objects: int and kcons. As described in the main text, the values for the calving and calf loss break points are constrained to be at least 24 step lengths away from the beginning and the end of the time-series and from one another. The object int specifies this constraint and in our analyses was set to equal 24. The main reason for this constraint is that a minimum number of data points are required in each section of the time-series to be able to estimate model parameters. The choice of the exact value for this constraint is somewhat arbitrary. The number needs to be small enough to allow for the detection of calves born close to the start of the time-series and for detecting calf loss events occurring shortly after birth or near the end of the time-series. However, the number needs to be large enough to ensure a sufficient amount of data within each section to accurately estimate parameters. In addition, as AIC selects a model based on the likelihood over the entire time-series, the models can only appreciably differ in AIC if the amount of data points in each section is large enough compared to the length of the time-series. We left int as an object that can be changed by the user, although int should be at the very least 1 and very small values might cause the function to return warnings and errors. Note that this constraint is in terms of observed step lengths. In fact, in our code break point values can only be at times for which there is data thereby ensuring that data points are available within each section of the time-series for parameter estimation. This restriction on break points could potentially explain why the IBM performs poorly with time-series containing large numbers of missing data points. For example, if calving occurs at a time where we have a missing location, the calving break point is likely to be assigned to the last non-missing location before the calving event. This approximation should be adequate if the data gap is small. However, as the data gap increases between the last available data point and the calving event, this approximation will become increasingly inadequate. The main reason we coded our models in this manner was to handle missing data points. There are likely ways to code these models without this restriction but the implementation of such code on time-series with large data gaps would be difficult.

The other additional input to mnll3M()is a numeric vector, kcons, which contains two values that specify the minimum and maximum values for parameters *k1* and *k2*. As mentioned in the main text, we constrained our values to be equivalent to the number of steps between three and six weeks. kcons is required in part because an interval is needed for the R function optimize(). In addition, constraining the values of *k1*and *k2* helps the IBM to accurately distinguish between the three models.

The function mnll3M() can be divided into five parts, three of which apply one of the three models to data. The first part of mnll3M() creates objects that will contain the results and calculates the sample size. The second part of mnll3M() fits M0 to data. To do so, it estimates the MLE of the scale parameter, represented in the code by b0, by taking the mean of the observed step lengths and uses b0 to directly calculate the mnll. The third part of mnll3M() fits M1 to data by first creating a vector, BP1ser, which contains all possible values for the calving break point, BP1,c. To get the mnll, the nll1() function is applied to all possible BP1,c values and the minimum nll value is selected. The BP1,c value that produced mnll is selected as the MLE of BP1,c. The MLE of the two other parameters, *b1* and *k1*, are estimated as in nll1(). The fourth part of mnll3M() fits M2 to data. As for M1­, it first identifies all possible combinations of BP2,c and BP2,l. To get the mnll and the MLEs for BP2,c and BP2,l, the nll2() function is applied to all possible combinations of break points. The minimum nll value is selected as the mnll and the two BP values producing mnll are selected as BP2,c and BP2,l. The two other parameters to estimate, *b2* and *k2* are estimated as in nll2(). The fifth part of mnll3M() uses the mnll values calculated for each model to calculate their AIC values. The model with the lowest AIC is selected as the best model. Finally, mnll3M() returns a list of three objects, which contains the MLE values of all parameters and the mnll and AIC of all models.

mnll3M <- function(SL, ti, tp, int, kcons){

resCA <- matrix(NA, 1, ncol=8)

colnames(resCA) <- c("n", "mnll_0", "mnll_1", "mnll_2",

"AIC0", "AIC1", "AIC2", "BM")

BPs <- data.frame(matrix(NA, 1, ncol=6))

colnames(BPs) <- c("BP1c", "BP2c", "BP2l", "iBP1c", "iBP2c",

"iBP2l")

mpar <- matrix(NA, 1, 5)

colnames(mpar) <- c("b0", "b1", "b2", "k1", "k2")

resCA[1] <- length(SL)

mpar[1] <- mean(SL) #b0

resCA[2] <- -sum(dexp(SL, 1/mpar[1], log=TRUE)) #mnll0

BP1ser <- int:(resCA[1]-int) #All possible BP1c

NLL1 <- lapply(BP1ser, nll1, SL=SL, ti=ti, kc=kcons)

MNLL1i <- which.min(NLL1)

resCA[3] <- NLL1[[MNLL1i]] #mnll1

BPs[4] <- BP1ser[MNLL1i] #BP1c MLE in terms of index of SL

BPs[1] <- as.character(tp[ti[BPs[[4]]]]) #BP1c in real time

mpar[2] <- mean(SL[1:BPs[[4]]]) #b1

mpar[4] <- optimize(nllk, kcons,

SLb=SL[(BPs[[4]]+1):resCA[1]],

tib=ti[(BPs[[4]]+1):resCA[1]], ba=mpar[2],

tiBP1=ti[BPs[[4]]])$minimum #k1

BP2ser <- combn(int:(resCA[1]-int), 2) #2 BPs combinations

BP2ser <- BP2ser[,diff(BP2ser) >= int]

BP2ser <- BP2ser[,diff(BP2ser) <= kcons[2]]

BP2ser <- split(t(BP2ser),1:ncol(BP2ser))

NLL2 <- lapply(BP2ser, nll2, SL=SL, ti=ti, kc=kcons)

MNLL2i <- which.min(NLL2)

resCA[4] <- NLL2[[MNLL2i]] #mnll2

BPs[5:6] <- BP2ser[[MNLL2i]] #mle of BP as index of SL

BPs[2] <- as.character(tp[ti[BPs[[5]]]]) #BP2c in real time

BPs[3] <- as.character(tp[ti[BPs[[6]]]]) #BP2l in real time

mpar[3] <- mean(SL[1:BPs[[5]]]) #b2

mpar[5] <- optimize(nllk, kcons,

SLb=SL[(BPs[[5]]+1):BPs[[6]]],

tib=ti[(BPs[[5]]+1):BPs[[6]]],

ba=mpar[3], tiBP1=ti[BPs[[5]]])$minimum #k2

resCA[5] <- 2*(resCA[2] + 1) #AIC0

resCA[6] <- 2*(resCA[3] + 3) #AIC1

resCA[7] <- 2*(resCA[4] + 4) #AIC2

resCA[8] <- which.min(resCA[,5:7])-1

return(list(resCA=resCA, BPs=BPs, mpar=mpar))

}

To apply the IBM to data, first run all of the code sections included in this appendix, then create the SL, ti, tp, int, and kcons objects, and finally run:

mnll3M(SL, ti, tp, int, kcons)

**References:**

Forbes, C., Evans, M., Hastings, N. & Peacock, B. (2011). *Statistical distributions*, 4th edn. John Wiley & Sons, Inc., Hoboken, New Jersey.

R Development Core Team. 2012. R: A Language and Environment for Statistical Computing. R Foundation for Statistical Computing, Vienna, Austria. ISBN 3-900051-07-0.

**Appendix 4:** Post-hoc Analyses of the Effects of Data Quality on Method Performance

We conducted post-hoc analyses to assess the effects of fix rate and data gaps on method performance. Fix rate is the number of successful GPS locations acquired by the radio-collar divided by the number of attempts (Frair *et al.* 2010) . We tested for these effects by using female caribou whose calf survival status was correctly predicted by both methods in 2011 (*n* = 7) and 2012 (*n* = 6) using the original data. For this subset of females, the mean fix rate for the original data was 96% (range: 94 - 98%). Nine females had calves surviving to four weeks of age while four lost calves. In all analyses, parturition predictions for both methods remained unchanged. We therefore focus primarily on how data quality affects predictions of neonate survival.

To assess the effects of fix rate, we randomly subsampled within each individual female to simulate fix rates of 90% down to 60% in 5% increments. To assess the effect of data gaps, we randomly removed intervals of one day, two non-consecutive days and two consecutive days from the post-calving period within the original time-series of each individual female. We then assessed the interacting effects of fix rate and data gaps. For all analyses, we ran 30 simulations across the data set of 13 individuals and calculated the mean accuracy rate (the percentage of correct predictions) for each method.

In general, the PBM was more robust to decreasing data quality. With decreasing fix rate, mean accuracy of the PBM stayed above 90% until fix rates fell below 80% while mean accuracy for the IBM fell below 90% with fix rates < 90% (Fig. A4.1). Data gaps had less effect than fix rate. Mean accuracy of the PBM was > 98% in all three scenarios (one day removed: 99% [SE: 2%]; two non-consecutive days: 98% [SE: 3%]; two consecutive days: 98% [SE: 3%]) while IBM mean accuracy was slightly lower (one day: 88% [SE: 5%]; two non-consecutive days: 94% [SE: 6%]; two consecutive days: 93% [SE: 6%]). Assessing the interacting effects of fix rate and data gaps, method performance was largely dictated by fix rate with the removal of days only slightly decreasing mean accuracy compared to fix rate alone (Fig. A4.2).

In all analyses, the majority (> 95%) of incorrect predictions resulted from surviving calves being misclassified (i.e. neonate survival was underestimated).

**References:**

Frair, J.L., Fieberg, J., Hebblewhite, M., Cagnacci, F., DeCesare, N.J. & Pedrotti, L. (2010). Resolving issues of imprecise and habitat-biased locations in ecological analyses using GPS telemetry data. *Philosophical Transactions of the Royal Society of London. Series B, Biological Sciences*, **365**, 2187–200.


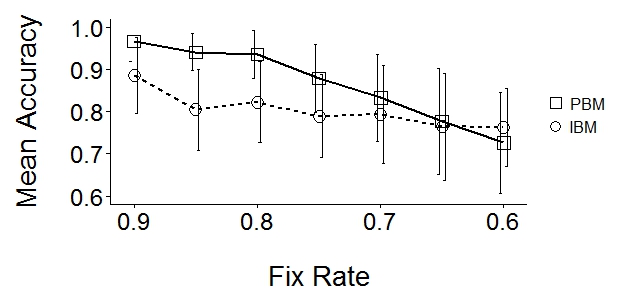


Figure A4.1: Predictive performance of the PBM and IBM when fix rate was reduced from 90% down to 60%. Thirty simulations were performed for each level of fix rate. Accuracy is the percentage of correct predictions of calf survival status (error bars = 1 SE).


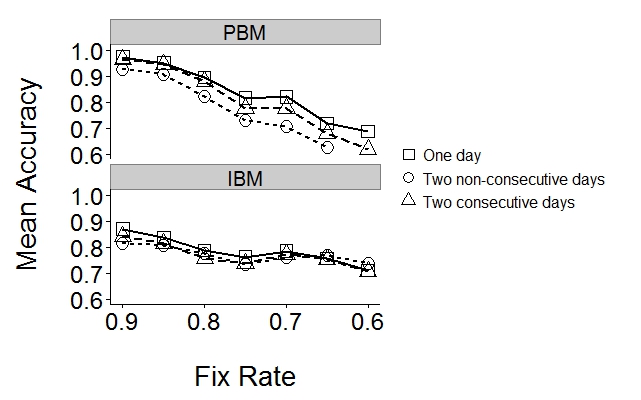


Figure A4.2: Predictive performance of the PBM (top) and the IBM (bottom) when fix rate is reduced and data gaps are present. Data gaps of one day, two non-consecutive days and two consecutive days were simulated in the post-calving interval of individual time-series. Thirty simulations were performed for each level of fix rate. Accuracy is the percentage of correct predictions of calf survival status. For clarity, error bars are not shown.
